# EEG-based emergency braking intention detection during simulated driving

**DOI:** 10.1186/s12938-023-01129-4

**Published:** 2023-07-01

**Authors:** Xinbin Liang, Yang Yu, Yadong Liu, Kaixuan Liu, Yaru Liu, Zongtan Zhou

**Affiliations:** College of Intelligence Science and Technology, National University of Defense Technology, Changsha, 410073 Hunan China

**Keywords:** Emergency braking intention, Electroencephalogram (EEG), Detection, Simulated driving, Brain-computer interface (BCI)

## Abstract

**Background:**

Current research related to electroencephalogram (EEG)-based driver’s emergency braking intention detection focuses on recognizing emergency braking from normal driving, with little attention to differentiating emergency braking from normal braking. Moreover, the classification algorithms used are mainly traditional machine learning methods, and the inputs to the algorithms are manually extracted features.

**Methods:**

To this end, a novel EEG-based driver’s emergency braking intention detection strategy is proposed in this paper. The experiment was conducted on a simulated driving platform with three different scenarios: normal driving, normal braking and emergency braking. We compared and analyzed the EEG feature maps of the two braking modes, and explored the use of traditional methods, Riemannian geometry-based methods, and deep learning-based methods to predict the emergency braking intention, all using the raw EEG signals rather than manually extracted features as input.

**Results:**

We recruited 10 subjects for the experiment and used the area under the receiver operating characteristic curve (AUC) and F1 score as evaluation metrics. The results showed that both the Riemannian geometry-based method and the deep learning-based method outperform the traditional method. At 200 ms before the start of real braking, the AUC and F1 score of the deep learning-based EEGNet algorithm were 0.94 and 0.65 for emergency braking vs. normal driving, and 0.91 and 0.85 for emergency braking vs. normal braking, respectively. The EEG feature maps also showed a significant difference between emergency braking and normal braking. Overall, based on EEG signals, it was feasible to detect emergency braking from normal driving and normal braking.

**Conclusions:**

The study provides a user-centered framework for human–vehicle co-driving. If the driver's intention to brake in an emergency can be accurately identified, the vehicle's automatic braking system can be activated hundreds of milliseconds earlier than the driver's real braking action, potentially avoiding some serious collisions.

## Background

The use of EEG in the driver status monitoring has received much attention in recent years. These studies can be divided into three categories: driver distraction, fatigue and intention detection [1]. The Driver intention studies are mainly about braking intention [2–7]. Before a driver brakes, there is a corresponding activity in the brain. By analyzing the driver's EEG signals while driving, it is possible to predict the upcoming braking behavior and activate the vehicle's emergency braking system in advance, which may prevent a collision. Haufe et al. designed an EEG-based emergency braking intention detection experiment, which was carried out on a simulated driving platform, and included two driving scenarios: emergency braking and normal driving [2]. Using linear discriminant analysis (LDA) classifier with an automatic shrinkage technique to classify manually extracted EEG features, the driver's emergency braking can be detected 130 ms before the actual braking behavior. Subsequently, Haufe et al. carried out a real-world driving experiment on a non-public test road. The experimental setup was similar to the simulated driving environment described above, and the results showed that the EEG features and detection results resembled those in the simulated environment [3]. Kim et al. investigated the neural correlates of emergency braking intentions under different driving situations. The experiments were conducted in a simulated driving environment. By collecting EEG data from different emergency braking scenarios and non-emergency braking scenarios and using the LDA to classify the features manually extracted, the results showed that the specific neural patterns of emergency braking intention could be well identified [4]. Hernandez et al. investigated the feasibility of detecting the emergency braking intention through EEG when the driver was under stress, workload, and fatigue. The experiments were conducted in a simulated driving environment containing both emergency braking and normal driving scenarios, and the EEG was collected simultaneously during the simulated driving. Support vector machines (SVM) and the convolutional neural network (CNN) were, respectively, used to classify EEG signals, and the results showed that both methods achieved an average classification accuracy of more than 70% under three different cognitive states [5]. Teng et al. proposed a model using the LDA with EEG spatial frequency features to detect the emergency braking intention. The experiment was conducted in a simulated driving environment with 12 subjects, and the results showed that a systematic accuracy of 94% could be achieved 420 ms after the onset of stimulus for an emergency braking [6]. To address the safety problem caused by certain recognition errors in detecting the driver’s emergency braking intention based on EEG, Bi et al. improved the accuracy and reduced the false alarm rate by combining the driver's EEG signals with the external information of the vehicle, which provides a new idea for human–vehicle co-driving [7].

By combing through the research on EEG-based detection of emergency braking intention, we found three points that can be further investigated. First of all, driving braking includes not only emergency braking (also called sharp braking), but also normal braking (also called soft braking, which is more common in the daily driving). At present, the studies on EEG-based braking intention detection mainly focus on detecting emergency braking from normal driving process, with less attention paid to the distinction between normal braking and emergency braking. Since emergency braking is usually closely related to driving safety, failure to accurately distinguish between these two types of braking may result in some serious traffic accidents. Second, current research on EEG-based emergency braking intention detection uses manually extracted features. And to manually extract EEG features, researchers need to have rich professional knowledge (e.g., they are very familiar with the experimental procedure, know very well the brain activity corresponding to different EEG features, etc.). However, since some EEG features may not be easily implemented for manual feature extraction or are not considered to be somehow associated with the driver's emergency braking intention, some important features may be omitted in the manual feature extraction process. Third, the current research on the detection of emergency braking intention mainly uses traditional machine learning methods. Although the literature [5] used the CNN classifier, however, it still used manually extracted features as input and did not take advantage of the powerful automatic feature learning capability of deep learning-based methods, nor did it achieve significantly better classification results than SVM.

To address the above three problems, this paper proposes a novel EEG-based experiment for driver’s emergency braking intention detection. We used a simulated driving platform to carry out the experiment to ensure safety. Relevant research has shown that the emergency braking intention in the simulated driving environment has similar EEG features to that in the real-world driving environment [3]. Our experiment included not only emergency braking and normal driving scenarios, but also normal braking scenarios. Subjects drove a simulated vehicle and completed a series of normal braking and emergency braking tasks according to the experimental requirements. In the emergency braking situation, subjects were required to apply the emergency brake immediately upon an external cue. In the normal braking situation, the subject performed the braking action spontaneously without external cues. The subject's EEG signals were recorded simultaneously. To overcome the problems associated with manual extraction of EEG features, we used the raw EEG signal with only simple filtering and baseline correction as the input to the classification algorithm. Due to the continuous advances in EEG signal processing algorithms, especially the successful application of Riemannian geometry-based and deep learning-based methods in brain-computer interfaces (BCIs), more and better options for decoding EEG signals are available. In this paper, we explored the use of traditional machine learning methods, Riemannian geometry-based methods and deep learning-based methods to detect the driver’s emergency braking intention [8–13].

The rest of the paper is organized as follows. The methods section contains the subjects, experimental setup, data preprocessing, classification algorithms, classification details, and evaluation metrics. The results section presents the emergency braking response time, EEG potential features and topographic maps, and classification results of different algorithms. In the discussion section, the results of the experiment are discussed and some limitations are pointed out. Finally, we give a conclusion.

## Results

### Emergency braking response time

**Fig. 1:**
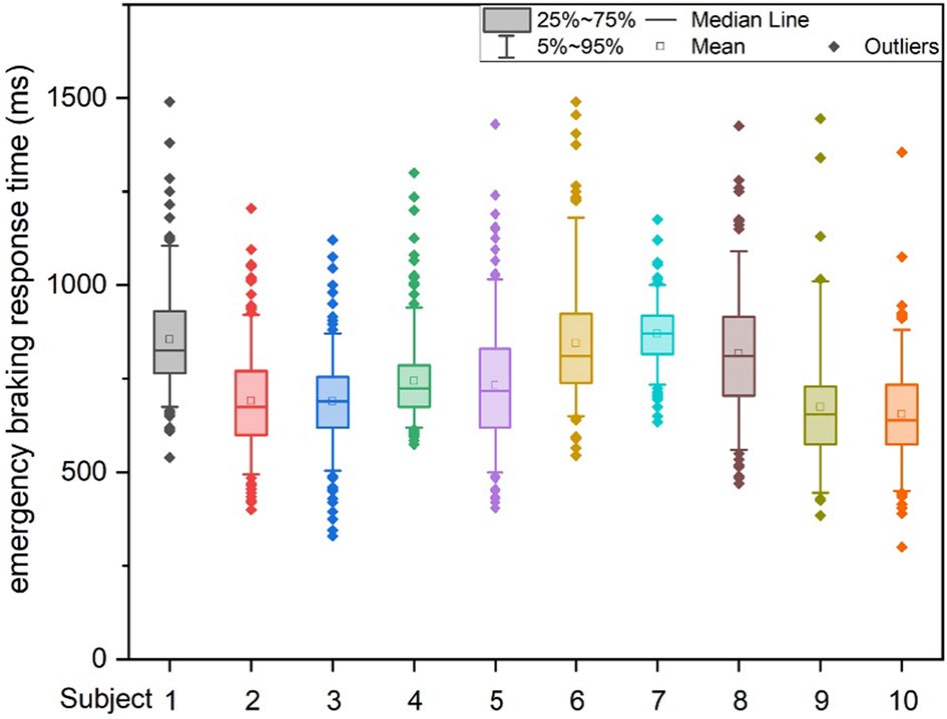
Distribution of emergency braking response time for each subject

The response time to emergency braking was defined as the duration from the time the brake lights of the lead vehicle came on until the subject started to depress the brake pedal in the emergency scenario. Emergency braking was a process in which the subject received a stimulus and responded to it. In our experiment, the mean emergency braking response time for the 10 subjects was 763 ± 157 ms, with a minimum of 300 ms and a maximum of 1490 ms. For all the subjects, the emergency braking response times were distributed as follows: P5 = 520 ms, P25 = 660 ms, P50 = 750 ms, P75 = 850 ms, and P95 = 1025 ms. Figure 1 shows the distribution of each subject’s emergency braking response times, including data distributions from 5 to 95%, 25% to 75%, as well as the mean, median and outliers. For each subject, the mean emergency braking response time was between 650 and 870 ms, and the median was between 640 and 870 ms, which were very close to each other.

### EEG potential features and topographic maps

**Fig. 2:**
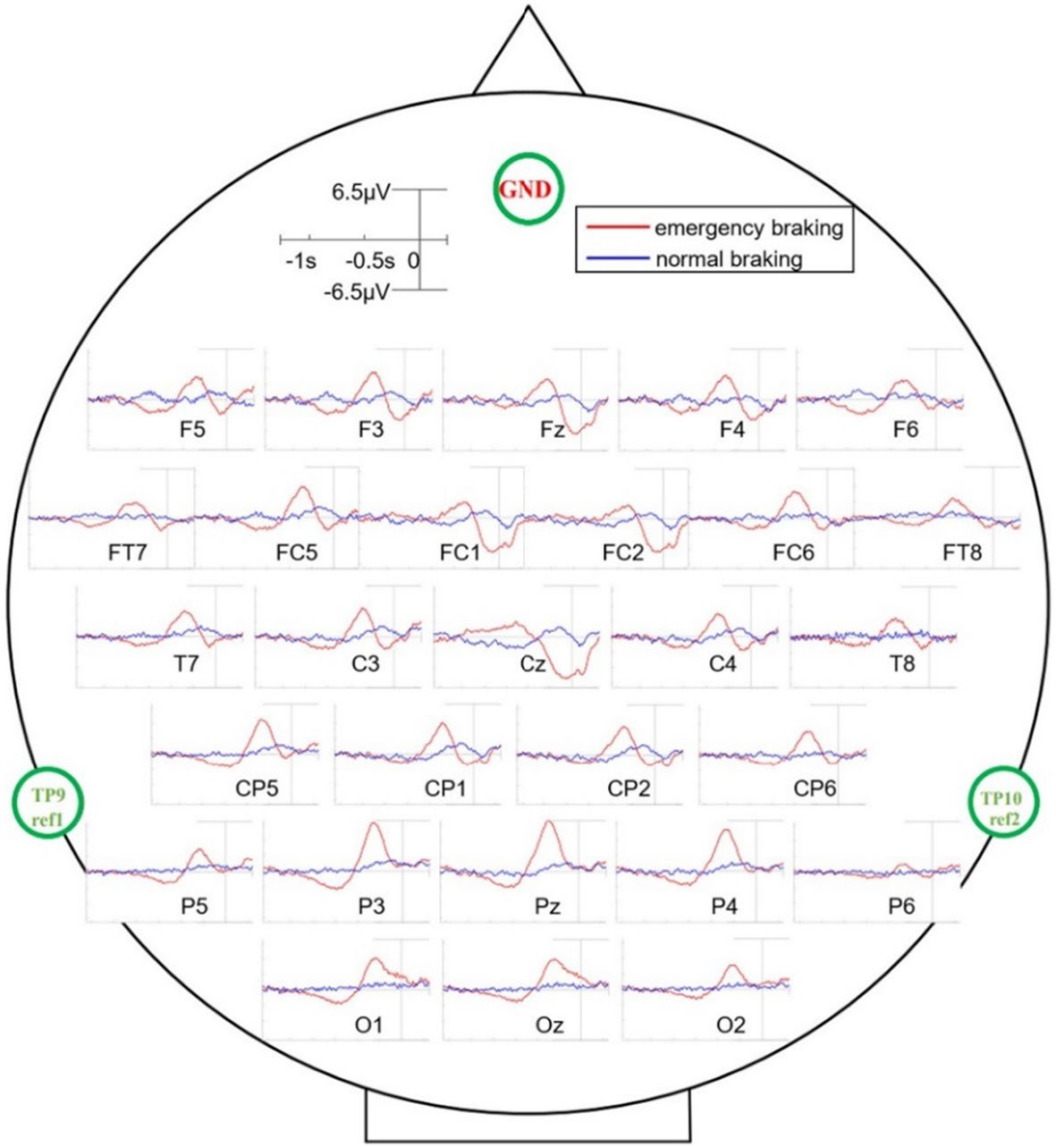
Grand-average EEG potential curves for different electrodes under emergency braking and normal braking scenarios. The red line represents results under emergency braking, while the blue line represents results under normal braking. The black vertical line at 0 indicates the onset of braking

Figure 2 shows the grand-average EEG potential curves for the 10 subjects under emergency braking and normal braking scenarios. The red line indicates the results under emergency braking scenarios while the blue line indicates the results under normal braking scenarios. The difference in EEG potentials between the two braking situations was significant: the range of variation in EEG amplitude was significantly greater during emergency braking (± 6.5 µV) than during normal braking (± 1.8 µV). In both braking scenarios, the EEG showed a general symmetry with respect to the longitudinal midline of the brain. In the parieto-occipital region (electrodes P3, Pz, P4, O1, Oz and O2), for the emergency braking situation, the EEG potential started to fall around −900 ms, reached a maximum negative offset at −600 ms (about −2 µV for P3, Pz, P4 and O1, −1.5 µV for Oz and −1 µV for O2), then started to rise and reached a maximum positive offset near −300 ms (about 6 µV for P3 and Pz, 5 µV for P4, 4 µV for O1 and Oz, and 3 µV for O2), whereas there was little change in the normal braking situation. Before and during emergency braking, a negative potential shift was observed in the frontal and central regions, which was most pronounced at Cz, with a negative shift starting at −400 ms and a maximum negative shift potential of −5.5 µV. A negative potential shift was also observed in the central region prior to normal braking onset, but it occurred earlier and to a lesser extent than in the emergency braking case, for example at electrode Cz, where the negative shift began at −900 ms with a maximum negative shift potential of −1.5 µV.

**Fig. 3:**
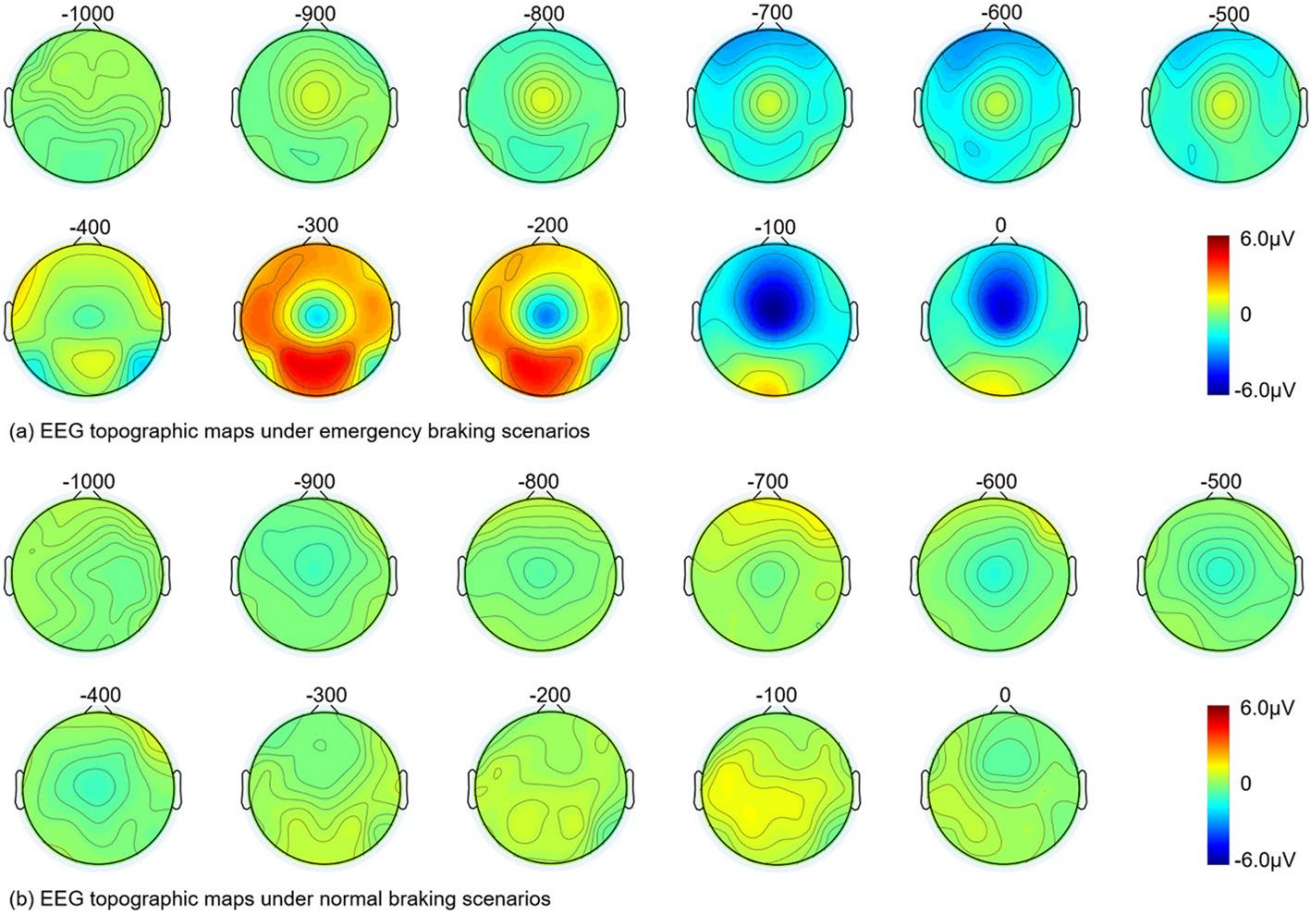
The grand-average EEG topographic maps from 1000 ms before braking to the onset of braking. Time 0 represents the onset of braking. Subfigure **a** is the result under emergency braking scenarios. Subfigure **b** is the result under normal braking scenarios

Figure 3 shows the grand-average EEG topographic maps for the 100 ms interval from 1000 ms before braking to the onset of braking. Subfigure (a) shows the results in the case of emergency braking and subfigure (b) shows the results in the case of normal braking. With Fig. 3, we were able to compare more visually the changes in EEG potentials in different brain regions before the onset of braking. In the emergency braking case, the potential in the occipital region increased from around –600 ms, reached a maximum (about 6 µV) around −300 ms, and then started to decrease, while no similar changes were found in the normal braking scenario. In the central region, the EEG in the emergency braking case started to decrease from about −400 ms and reached a minimum (about −5.5 µV) at about −100 ms, whereas in the normal braking case there was a smaller negative shift from −900 ms to −400 ms (the maximum negative shift was −1.5 µV and occurred at −500 ms). Overall, a wider range of EEG potential amplitudes and a more dramatic course of positive and negative potential changes were observed under emergency braking compared to normal braking.

### C. Classification performance

**Fig. 4:**
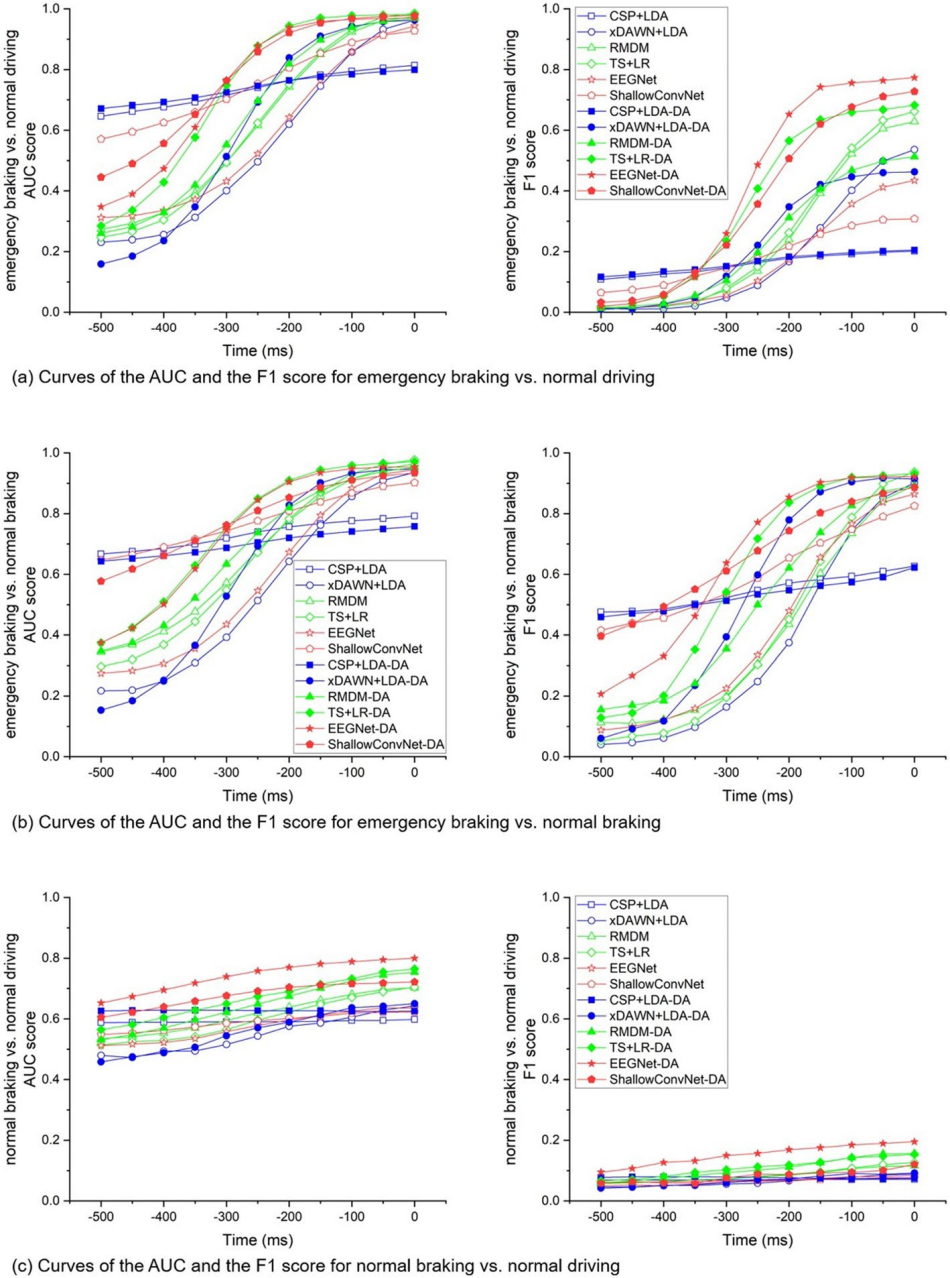
AUC and F1 score curves. The algorithm with “-DA” indicates that its training set used data augmentation. Time 0 represents the onset of braking. The curves are the test results of intercepting data on the test set by a sliding window of 1500 ms in length and 50 ms in step size, with the AUC and F1 score corresponding to the end point of the sliding window

Subfigures (a), (b) and (c) of Fig. 4 show the AUC and F1 score curves for different algorithms under emergency braking vs. normal driving, emergency braking vs. normal braking and normal braking vs. normal driving, respectively. The algorithm with “-DA” indicates that it used data augmentation for the training set. The 0 on the time axis represents the onset of braking. The curves in the figure are the test results of data intercepted on the test set through a sliding window of 1500 ms in length and 50 ms in step size, with the AUC and F1 score corresponding to the end point of the sliding window. Tables 1 and 2 show the AUCs and F1 scores for the different algorithms at −200 ms, −100 ms and the moment of braking onset, respectively. We got the following results.

- Subjects’ emergency braking intention was well detected from normal driving based on the EEG signal. 200 ms before the onset of braking, the AUCs of both TS + LR-DA and EEGNet-DA reached 0.94 and the F1 score of EEGNet-DA was 0.65; 100 ms before braking, the AUC of TS + LR-DA was 0.98 and the F1 score of EEGNet-DA was 0.76.
- For the two different braking modes, emergency braking and normal braking can be accurately distinguished based on EEG signals. 200 ms before the onset of braking, the AUC of EEGNet-DA was 0.91 and F1 score was 0.85, and 100 ms before the onset of braking, TS + LR-DA achieved an AUC of 0.96 and an F1 score of 0.92.
- Based on EEG, it was difficult to detect normal braking from normal driving. At −200 ms, −100 ms and the moment of braking onset, the optimal F1 scores of algorithms were only 0.17, 0.18 and 0.20, respectively.
- The classification performance of the algorithms was different in the experiment. For the detection of emergency braking intention, the Riemannian geometry-based TS + LR algorithm and the deep learning-based EEGNet show a greater advantage in the time frame of 200 ms before the onset of braking, while the CSP + LDA algorithm performs poorly.
- Augmentation of the training data can improve the prediction performance of certain algorithms. For example, 200 ms before the onset of braking, the AUCs and F1 scores improved by 0.3 and 0.48 for EEGNet and 0.19 and 0.31 for TS + LR for emergency braking vs. normal driving, respectively; for emergency vs. normal braking, the AUCs and F1 scores improved by 0.19 and 0.4 for xDAWN + LDA and 0.24 and 0.37 for EEGNet, respectively. However, for the CSP + LDA algorithm, the augmentation of the training data had almost no effect in our experiment.

**Table 1:** AUCs of algorithms under different cases and the results are expressed as mean ± standard deviation

Algorithms	Emergency braking vs. Normal driving			Emergency braking vs. Normal braking			Normal baking vs. Normal driving		
	−200 ms	−100 ms	0	−200 ms	−100 ms	0	−200 ms	−100 ms	0
CSP + LDA	0.76 ± 0.14	0.79 ± 0.15	0.81 ± 0.15	0.76 ± 0.13	0.78 ± 0.14	0.79 ± 0.15	0.59 ± 0.09	0.59 ± 0.09	0.60 ± 0.09
xDAWN + LDA	0.62 ± 0.21	0.86 ± 0.15	0.96 ± 0.04	0.64 ± 0.17	0.86 ± 0.12	0.94 ± 0.08	0.58 ± 0.08	0.61 ± 0.09	0.64 ± 0.12
RMDM	0.74 ± 0.16	0.92 ± 0.09	0.97 ± 0.04	0.78 ± 0.14	0.91 ± 0.10	0.96 ± 0.05	0.64 ± 0.11	0.68 ± 0.11	0.70 ± 0.12
TS + LR	0.75 ± 0.16	0.93 ± 0.07	**0.99 ± 0.02**	0.78 ± 0.17	0.93 ± 0.09	**0.98 ± 0.03**	0.62 ± 0.11	0.67 ± 0.11	0.71 ± 0.12
EEGNet	0.64 ± 0.19	0.86 ± 0.14	0.94 ± 0.05	0.67 ± 0.12	0.88 ± 0.06	0.94 ± 0.03	0.60 ± 0.08	0.63 ± 0.08	0.64 ± 0.09
ShallowConvNet	0.81 ± 0.09	0.89 ± 0.07	0.93 ± 0.05	0.81 ± 0.08	0.87 ± 0.08	0.90 ± 0.07	0.60 ± 0.07	0.62 ± 0.07	0.62 ± 0.07
CSP + LDA-DA	0.76 ± 0.14	0.78 ± 0.14	0.80 ± 0.14	0.72 ± 0.16	0.74 ± 0.16	0.76 ± 0.16	0.63 ± 0.07	0.63 ± 0.07	0.63 ± 0.07
xDAWN + LDA-DA	0.84 ± 0.15	0.94 ± 0.08	0.96 ± 0.04	0.83 ± 0.13	0.93 ± 0.07	0.95 ± 0.05	0.59 ± 0.11	0.64 ± 0.11	0.65 ± 0.12
RMDM-DA	0.82 ± 0.11	0.94 ± 0.07	0.97 ± 0.04	0.82 ± 0.14	0.91 ± 0.09	0.95 ± 0.06	0.67 ± 0.13	0.72 ± 0.13	0.75 ± 0.13
TS + LR-DA	**0.94 ± 0.05**	**0.98 ± 0.02**	0.98 ± 0.02	**0.91 ± 0.10**	**0.96 ± 0.05**	0.97 ± 0.04	0.69 ± 0.13	0.73 ± 0.13	0.76 ± 0.12
EEGNet-DA	**0.94 ± 0.05**	0.97 ± 0.03	0.97 ± 0.03	**0.91 ± 0.06**	0.95 ± 0.04	0.95 ± 0.04	**0.77 ± 0.09**	**0.79 ± 0.10**	**0.80 ± 0.10**
ShallowConvNet-DA	0.92 ± 0.05	0.97 ± 0.03	0.98 ± 0.02	0.85 ± 0.10	0.91 ± 0.08	0.93 ± 0.06	0.70 ± 0.09	0.72 ± 0.10	0.72 ± 0.10

Algorithms with “-DA” mean that their training set used data augmentation; −200 ms means 200 ms before the onset of braking

Best results are highlighted in bold

**Table 2:** F1 scores of algorithms under different cases and the results are expressed as mean ± standard deviation

Algorithms	Emergency braking vs. normal driving			Emergency braking vs. normal braking			Normal baking vs. normal driving		
	−200 ms	−100 ms	0	−200 ms	−100 ms	0	−200 ms	−100 ms	0
CSP + LDA	0.18 ± 0.13	0.19 ± 0.13	0.20 ± 0.13	0.57 ± 0.18	0.59 ± 0.18	0.63 ± 0.19	0.07 ± 0.02	0.07 ± 0.02	0.07 ± 0.02
xDAWN + LDA	0.17 ± 0.15	0.40 ± 0.23	0.54 ± 0.18	0.38 ± 0.18	0.75 ± 0.17	0.90 ± 0.08	0.07 ± 0.03	0.08 ± 0.03	0.09 ± 0.05
RMDM	0.24 ± 0.20	0.52 ± 0.27	0.63 ± 0.24	0.44 ± 0.20	0.73 ± 0.21	0.89 ± 0.11	0.08 ± 0.04	0.11 ± 0.05	0.12 ± 0.06
TS + LR	0.26 ± 0.23	0.54 ± 0.25	0.66 ± 0.17	0.45 ± 0.25	0.79 ± 0.18	**0.94 ± 0.05**	0.09 ± 0.04	0.11 ± 0.06	0.13 ± 0.07
EEGNet	0.17 ± 0.17	0.36 ± 0.20	0.43 ± 0.16	0.48 ± 0.2	0.77 ± 0.24	0.86 ± 0.19	0.07 ± 0.02	0.08 ± 0.04	0.08 ± 0.05
ShallowConvNet	0.22 ± 0.13	0.29 ± 0.15	0.31 ± 0.14	0.65 ± 0.17	0.75 ± 0.15	0.83 ± 0.10	0.07 ± 0.02	0.07 ± 0.03	0.08 ± 0.02
CSP + LDA-DA	0.18 ± 0.12	0.20 ± 0.13	0.21 ± 0.13	0.55 ± 0.21	0.57 ± 0.21	0.62 ± 0.20	0.08 ± 0.02	0.07 ± 0.02	0.08 ± 0.02
xDAWN + LDA-DA	0.35 ± 0.20	0.45 ± 0.18	0.46 ± 0.18	0.78 ± 0.13	0.9 ± 0.06	0.91 ± 0.07	0.07 ± 0.03	0.09 ± 0.04	0.09 ± 0.05
RMDM-DA	0.31 ± 0.23	0.47 ± 0.24	0.51 ± 0.23	0.62 ± 0.21	0.83 ± 0.14	0.89 ± 0.11	0.11 ± 0.07	0.14 ± 0.08	0.16 ± 0.09
TS + LR-DA	0.57 ± 0.19	0.66 ± 0.17	0.68 ± 0.17	0.84 ± 0.12	**0.92 ± 0.06**	0.93 ± 0.06	0.12 ± 0.06	0.14 ± 0.08	0.15 ± 0.08
EEGNet-DA	**0.65 ± 0.18**	**0.76 ± 0.15**	**0.77 ± 0.13**	**0.85 ± 0.11**	**0.92 ± 0.07**	0.92 ± 0.07	**0.17 ± 0.09**	**0.18 ± 0.10**	**0.20 ± 0.10**
ShallowConvNet-DA	0.51 ± 0.19	0.68 ± 0.14	0.73 ± 0.12	0.74 ± 0.12	0.84 ± 0.10	0.89 ± 0.08	0.09 ± 0.04	0.09 ± 0.06	0.12 ± 0.08

Algorithms with “-DA” mean that their training set used data augmentation; −200 ms means 200 ms before the onset of braking

Best results are highlighted in bold

## Discussion

In the following, we focus on three aspects of emergency braking response time, EEG features of the two braking modes and classification algorithms, and conclude with a description of the limitations of this study.

For the emergency braking response time, the mean value of the 10 subjects in the experiment was 763 ms, and the mean value for each subject ranged from 650 to 870 ms. The Kruskal–Wallis rank-sum test showed significant differences in the mean reaction time across subjects (p-value less than 0.05). In the literature [2], the median emergency braking response time was 665 ms. The mean response time was 720 ms in [3], 718 ms in [5] and 833.7 ms in [7]. Our results were generally consistent with related studies.

For the EEG signals during the braking process, we found very significant differences between the two braking modes, emergency braking and normal braking. The emergency braking process consisted of starting with the brake lights of the lead vehicle coming on, to the subject noticing this stimulus and forming a cognitive decision, and then making the decision to immediately release the gas pedal and apply the brake. During this process, the brake lights of the lead vehicle came on as a relatively strong and less frequent visual stimulus, which was continuously reinforced as the distance to the lead vehicle rapidly decreased, similar to the “oddball” stimulus in the P300-based BCIs [14, 15]. A very pronounced positive potential shift was observed at the location of the parieto-occipital region near 300 ms before the onset of emergency braking, which we consider as a P300 component. In contrast, we did not find a P300-like component in the parieto-occipital region during normal braking, because the braking process was made spontaneously by the subject without external stimulus similar to the emergency braking scenario. In the emergency braking situation, a negative offset potential in the frontal–central region (especially at electrode Cz) related to the movement planning and execution process was observed both before and during the right foot switching from the gas pedal to the brake pedal [2, 4, 16]. Under normal braking, the right foot of the subject also completed the action of releasing the gas pedal and then stepping down the brake pedal. These two actions were the same as those under emergency braking, but they were both performed in a relaxed state, lacking the sense of urgency under emergency braking scenarios, and the corresponding potential changes in brain regions associated with movement were not as large as those under emergency conditions. Furthermore, normal braking was an spontaneous braking behavior controlled by the subject, who had sufficient preparation time before braking, which might explain the negative potential shift in the central region of the brain under normal braking to be earlier than under emergency braking [17]. In the normal driving scenario, there were also visual stimuli with real-time changes in the surroundings and the movement preparation/execution of acceleration/deceleration maneuvers. However, our experiments suggested that the orderly superposition and combination of these related EEG features and the driver's state of tension may be the key to reflect the emergency braking intention.

For the classification algorithms, we selected six commonly used EEG decoding algorithms, CSP + LDA, xDAWN + LDA, RMDM, TS + LR, EEGNet and ShallowConvNet, which represent traditional machine learning methods, Riemannian geometry-based methods and deep learning-based methods. For emergency braking vs. normal driving and emergency braking vs. normal braking, TS + LR had an advantage in the AUC (which was more pronounced when the training set was not augmented with data), and EEGNet has an advantage in the F1 score (the training set with DA) when choosing the time period −200 ms to 0 relative to the onset of braking. If we can predict the driver's intention to brake in an emergency situation 200 ms before the actual braking action, it means that when the vehicle is traveling at 90 km/h, the braking distance will be reduced by 5 m, that is, the length of a car. Deep learning algorithms like EEGNet and ShallowConvNet typically require large datasets, and the classification results are often poor when there are few training samples. For example, 100 ms before the onset of braking, for emergency braking vs. normal driving, the AUCs of EEGNet and ShallowConvNet were 0.86 and 0.89, and F1 scores were 0.36 and 0.29, respectively, when no DA was used in the training set. Neither the AUCs nor the F1 scores were as good as the RMDM and TS + LR. To this end, we used DA on the training set to fully exploit the performance of the deep learning methods. After DA, the AUCs of both EEGNet and ShallowConvNet were 0.97 (an increase of 0.11 and 0.08 compared to pre-DA), which were comparable to the result of TS + LR (0.98) and outperformed the other algorithms. The F1 scores of these two algorithms were 0.76 and 0.68 (an increase of 0.4 and 0.39), respectively, which exceed the other algorithms. For both EEGNet and ShallowConvNet, the AUCs and F1 scores were significantly improved, and their corresponding standard deviations were reduced, indicating that the robustness of the algorithm was enhanced with the improved performance. EEG datasets usually have a small sample size, which is not enough to train a deep network, so CNN models used for EEG classification usually have a small number of layers [18]. Both EEGNet and ShallowConvNet used in this study are shallow-layered neural networks. Considering the practical application of the system, we also need to calculate the time consumption of different algorithms. Methods based on Riemannian geometry usually require a large number of complex calculations, which are currently performed only on CPUs and require more time consumption. Deep learning-based methods also require a large number of calculations, but these calculations can be accelerated using GPUs, thus saving the training and execution time. In this experiment, the time required for one detection of emergency braking intention by CSP + LDA, xDAWN + LDA, RMDM, TS + LR, EEGNet and ShallowConvNet was about 1.8 ms, 2.5 ms, 5.6 ms, 6.2 ms, 5.6 ms and 5.6 ms, respectively, which fully satisfied the online requirements. Our experimental results were not easy to compare with related studies, because most of these studies were conducted in different experimental environments, and the evaluation metrics were different.

Although our experiment shows the feasibility of emergency braking intention detection based on EEG, there are still some limitations that need to be addressed in the future. The first limitation is that the experiment was conducted on a simulated platform with an ideal driving environment. Although it has been shown that the driver’s EEG signal during emergency braking in a real-world scenario has similar characteristics to those in a simulated scenario [3], we believe that the experiment in a real-world scenario still deserve focused attention. On one hand, the fully immersive driving experience in the real world may induce stronger brain activity. On the other hand, there are more disturbances in real-world driving, which may contaminate the EEG signals and thus affect the classification performance. The second limitation is that the subjects of the experiment were 10 graduate students aged 22–36 years, 8 of whom were male and 2 were female. The number of subjects participating in the experiment was relatively small and did not cover all age groups, and there was a certain bias in the ratio of male to female. In the future, we intend to conduct a larger experiment with more subjects of different ages and to keep the ratio of males to females roughly the same. The third limitation is that we currently have difficulty in detecting normal braking from normal driving, with none of the six algorithms giving satisfactory results. If the normal braking behavior of the driver can be predicted in advance, the vehicle will be able to take corresponding actions in advance according to the driver's intention, resulting in a more friendly human–vehicle interaction. The fourth limitation is that although relatively good classification results have been obtained for both emergency braking vs. normal driving and emergency braking vs. normal braking, they do not yet meet the requirements of practical applications. For example, the highest F1 score for the classification of emergency braking vs. normal driving is only 0.65 (the corresponding precision and recall of 0.62 and 0.63, respectively) 200 ms before the onset of braking, which clearly does not meet the requirements for practical system applications. The effectiveness of emergency braking intention detection is directly related to driving safety and needs to be treated with care. To realize practical applications of this system, we believe that there are two main ideas to focus on. One idea is to continue researching approaches based on EEG signals alone, including more convenient EEG acquisition devices, better filters, more reliable classification algorithms, etc. The other is to combine a system with other methods, such as the emergency braking intention detection system that combines EEG signals with external information, as proposed in [7].

## Conclusion

In this paper, we designed and conducted the experiment on a simulated driving platform for emergency braking intention detection based on driver’s EEG signals. The EEG potential features and topographic maps as well as classification results showed that it was feasible to detect emergency braking from normal driving and normal braking in advance. There are still some limitations in the current experiment, and in the future, we will focus on conducting experiments in real-world driving scenarios and developing advanced EEG decoding algorithm to solve the proposed problem. Due to the safety issues involved, the implementation of EEG-based emergency braking intention detection system in the real world will require some time for further in-depth research, but the application of this system in virtual scenarios (e.g., games and metaverse) may come soon.

## Methods

### Subjects

A total of 10 subjects (aged 22–36 years, 8 males and 2 females) participated in the experiment. All subjects provided written consent prior to the experiment. All procedures of the study were in accordance with the 1964 Declaration of Helsinki. The subjects were all graduate students at our university, had a driver's license, and had at least 2 years of driving experience. All subjects had normal or corrected normal vision and were right handed. They all declared no history of brain disease or mental disorders. The experiment was completed in 1 day, with each subject getting adequate sleep and not taking any drugs or alcohol for 3 days prior to the experiment. The purpose of the experiment and the experimental procedure were explained to the subjects before the start of the experiment, and each subject was given 30 min of practice time before the experiment formally began to ensure that they were proficient in the experimental task. Subjects could end the experimental task at any time during the experiment, which would not carry any penalty. If the subject successfully completed the experiment, he would receive 400 RMB as payment.

### Experimental setup

**Fig. 5:**
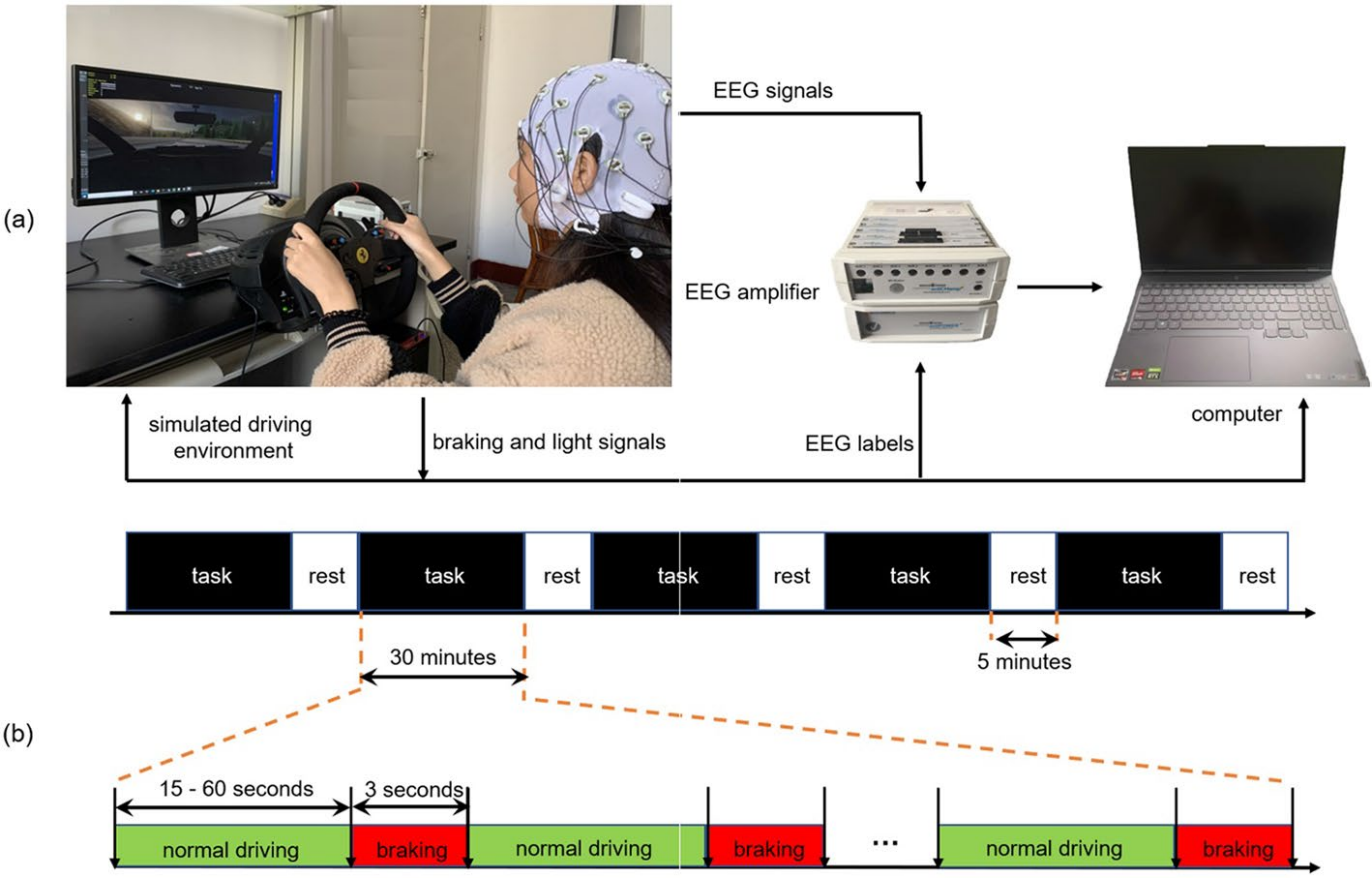
An illustration of the experimental setup. Subfigure **a** shows the composition of the simulated driving platform in the experiment. Subfigure **b** is the illustration of the experimental sequence. Note that the braking includes normal braking and emergency braking

Figure 5 illustrates the framework of the driving simulation experiment platform, which consists of an EEG acquisition device (a set of electrodes, an EEG cap and an EEG amplifier, ActiCHamp, Brain Products, Germany), a driving simulator (a steering wheel, a brake pedal, a gas pedal and a driver’s seat, T300 of Thrustmaster, France) and two computers. The Car Learning to Act (CARLA) platform was used to build the simulated driving environment, which was presented through a 24-inch LED display with a resolution of 1920 × 1080 [19]. Subjects used the driving simulator to control the simulated vehicle and to complete the tasks set in the experiment. EEG acquisition equipment was used to record the scalp EEG signals of the subject while driving. To distinguish the EEG data in different driving scenarios, we used CARLA's application program interface (API) to read vehicle status information in real time to achieve automatic tagging of EEG signals. The simulated system was refreshed 60 times per second, so there was a maximum delay of 16.7 ms for the status information recorded through the API. The computers served as a user interface to present the driving simulation environment, and as a data recording device to store the EEG signals.

In the simulated experiment environment, a total of two vehicles were included, one controlled by the subject to complete different experimental tasks as required, and the other controlled by the instructor as a lead vehicle. Two braking scenarios were set up in the experiment: emergency braking and normal braking. In the emergency braking scenario, the lead vehicle in front was traveling at 90 km/h. The subject-controlled vehicle followed the lead vehicle, stayed in the same lane, and maintained a distance of 6–12 m from the lead vehicle. The lead vehicle would randomly slow down sharply every 15–60 s and alert the following vehicle by flashing its brake lights. To avoid a collision, the subject needed to emergency brake the following vehicle under their control as soon as they saw the brake lights of the lead vehicle come on. During this process, two moments were recorded as marker information for EEG signals: one was the moment when the brake lights of the lead vehicle came on and the other was the moment when the subject started to depress the brake pedal. After decelerating for 3 s, the lead vehicle began to accelerate and again maintained 90 km/h. The subject, driving the simulated vehicle, continued to follow the lead vehicle and maintained a distance of 6 to 12 m. The driving speed and the distance from the lead vehicle were displayed in real time in the user window, and the subject could adjust the speed of the following vehicle under his control accordingly. In the normal braking scenario, the experiment was set up similarly to the emergency braking scenario, except that the lead vehicle no longer performed random emergency deceleration, but maintained a speed of 90 km/h. The subject drove the same vehicle on the same road as in the emergency braking scenario, following the lead vehicle in front and maintaining a distance of 6 to 12 m from it. Every 15–60 s, the subject spontaneously and normally braked the vehicle he was driving. Three seconds after the subject depressed the brake pedal, he accelerated the vehicle, followed the lead vehicle in front, and again maintained a distance of 6 to 12 m from it. The moment when the subject depressed the brake pedal captured through the API of the simulation platform was used as a marker for the EEG signal. As shown in Fig. 5b, each subject was required to complete the same driving task five times, with each task lasting 30 min and the subject resting for 5 min after each task was completed.

### Data preprocessing

The EEG amplifier and electrodes were used to acquire EEG data from the subject during driving. A total of 31 electrodes were used, arranged according to the international 10–20 system, of which 28 electrodes (Fz, F3, F5, FT7, FC5, FC1, T7, C3, CP5, CP1, Pz, P3, P5, O1, Oz, O2, P4, P6, CP6, CP2, FC2, FC6, FT8, Cz, C4, T8, F4 and F6) were used to record EEG signals, one electrode (FPz) was the ground electrode and the remaining two electrodes (TP9 and TP10) were used as reference electrodes. The resistance of the electrodes was adjusted so that they all remained below 10 kΩ. The sampling rate was set to 200 Hz, and a 50 Hz notch filter (second-order Butterworth filter) was used to remove the interference from the power supply frequency. A finite impulse response (FIR) bandpass filter (zero-phase, non-causal FIR filter with Hamming window) with a low frequency of 0.53 Hz and a high frequency of 45 Hz was used to filter the collected EEG signals. We used the FIR filter recommended in [20], mainly because that it is easier to control, always stable, has a clear passband and can be corrected to zero-phase without additional calculations. The EEG signals corresponding to the three different scenarios of normal driving, normal braking and emergency braking needed to be extracted separately. For the normal braking and emergency braking scenarios, we intercepted the EEG data from 2500 ms before to 500 ms after the moment when the subject started to depress the brake pedal as the target epoch. Epochs of normal driving were intercepted by sliding a window of 3000 ms in length and 500 ms in step size over the EEG signals collected while the subject was driving normally, and ensuring that the window used above was at least 3000 ms away from any braking behavior of the subject. After preprocessing, each subject had 208 ± 50 emergency braking epochs, 126 ± 37 normal braking epochs and 6650 ± 2298 normal driving epochs, as shown in Table 3.

**Table 3:** Number of samples per subject after data preprocessing

Subject	Emergency braking	Normal braking	Normal driving
S1	96	104	3923
S2	190	130	5033
S3	200	209	4811
S4	167	118	4110
S5	268	166	7185
S6	221	93	11093
S7	257	88	9223
S8	245	134	7207
S9	223	109	7465
S10	212	112	6450
Mean ± Std	208 ± 50	126 ± 37	6650 ± 2298

### Classification algorithms

To more systematically and comprehensively analyze the effect of EEG-based driver emergency braking intention detection, we explored six representative EEG decoding algorithms that are commonly used in BCIs, namely, the common spatial pattern filter combined with a linear discriminant analysis classifier (CSP + LDA) [21, 22], the xDAWN filter combined with a linear discriminant analysis classifier (xDAWN + LDA)[23, 24], the Riemannian minimum distance to mean classifier (RMDM) [9], the tangent space projection of covariance matrix with logistic regression classifier (TS + LR) [25, 26], EEGNet [12] and ShallowConvNet [11]. The above six algorithms can be grouped into three categories: traditional machine learning classifiers (CSP + LDA, xDAWN + LDA), Riemannian geometry-based classifiers (RMDM, TS + LR) and deep learning-based classifiers (EEGNet, ShallowConvNet). They all used the raw EEG signals with simple filtering and baseline correction as input in the experiment. As traditional classification algorithms, CSP + LDA is widely used in spontaneous activity-based BCIs, while xDAWN + LDA has a wide range of applications in event related potential (ERP)-based BCIs, where CSP and xDAWN act as adaptive filters that serve to improve the signal-to-noise ratio and reduce its feature dimensionality of the raw EEG signal [27]. EEG decoding algorithms based on Riemannian geometry change some conventions in traditional methods. Instead of trying to estimate the optimal filters corresponding to different types of EEG signals, they directly map the EEG signal onto a geometric space with suitable classification metrics. Although Riemannian geometry-based approaches have been applied in the field of EEG decoding for a short time, they have already achieved great success, such as winning five recent international BCI competitions [9]. For each given class, the RMDM algorithm estimates a centroid based on the chosen metric, and for each new point, its class is determined based on the nearest centroid. RMDM requires no manual parameter tuning and has a high classification accuracy, making it often used as a benchmark to measure the performance of other algorithms [8]. The TS + LR algorithm further projects the covariance matrix obtained in the RMDM algorithm onto the tangent space and then applies the logistic regression classifier to the projected data. Encouraged by the great success of deep learning in the field of computer vision, deep learning-based approaches, especially CNNs, have gained widespread attention in EEG signal classification [13, 28]. Deep learning-based algorithms have the ability to automatically learn features from training data, giving them end-to-end capabilities that make them ideal for online decoding of EEG signals. EEGNet and ShallowConvNet are CNNs specifically designed for EEG decoding, and application results in different types of BCI paradigms have shown their excellent classification performance [11, 12, 26, 29–31].

### Classification details

In this paper, we focus on whether the driver’s emergency braking intention can be detected in advance by the EEG signal. Therefore, the classification of emergency braking vs. normal driving and emergency braking vs. normal braking were used as the main objects of study. In addition, we also classified normal braking vs. normal driving as a comparison of emergency braking vs. normal driving. We referred to the literature [2] and [3] for the division of the training and test sets, and used the data in the first half of the experiment as the training set and the data in the second half as the test set. The training set for each subject contained 104 ± 25 emergency braking epochs, 63 ± 18 normal braking epochs and 3325 ± 1149 normal driving epochs, and the number of the three categories in the test set was equal to the training set. The training set was used to train the classifier, while the reported results were obtained based on the test set only. Compared to the cross-validation scheme, our sequential division approach reduces the classification performance of the system, but from a practical point of view, it makes more sense to use only the first part of the data because we cannot train the classifier with data that has not yet been generated.

Since these three types of EEG epochs were very different in number, they should not be used directly to train the classifier. We adopted two strategies to refine the training set. The first one was to use the one with the least number of EEG epochs among the three categories as the benchmark, and to make a random selection among the remaining two categories to keep the number of the three categories equal (as shown in Table 4). In the new training set, for both emergency braking and normal braking segment, we selected EEG data of 1500 ms before the onset of braking. The focus of this study was to predict braking intention, therefore, we used only EEG data before the onset of braking and not after. As for the segment of normal driving, the length of the normal driving epoch obtained in the data preprocessing section above was 3000 ms, and we used a window of 1500 ms in length to randomly intercept a segment on it, with only one segment per epoch. The segment-wise baseline correction was performed by subtracting the average EEG data for the first 100 ms of the extraction interval. In the second strategy we used the sliding window, which is a common method of EEG data augmentation (DA) [32], to augment the number of emergency braking and normal braking epochs to be consistent with normal driving (as shown in Table 4). The sliding window was 1500 ms in length, and its end point slid in the range of −200 ms to 200 ms relative to the onset of braking. Based on the length of the sliding range (400 ms) and the growth factor of the sample size, we determined the sliding step, for example, if the sample size needed to be increased to 40 times the original size, the sliding step was 10 ms (400/40 = 10). For the normal driving segment, we used a 1500 ms window to randomly intercept a segment of EEG data in the normal driving epoch obtained in the above data preprocessing section, with only one segment per epoch. Similarly, the segment-wise baseline correction was performed for the augmented training set. For the choice of sliding time range, we refer to the relevant literature on driver emergency braking response time [2–4, 6]. Using the sliding window for DA not only significantly increased the number of training samples (emergency braking augmented by about 32 times and normal braking augmented by about 53 times), but also expanded the input time range of the training data. Note that the above two methods were only for the training set, and no changes were made to the test set.

**Table 4:** Number of samples per subject in the training set under different strategies

Subject	Emergency braking			Normal braking			Normal driving		
	Original	Strategy 1	Strategy 2	Original	Strategy 1	Strategy 2	Original	Strategy 1	Strategy 2
S1	48	48	1962	52	48	1962	1962	48	1962
S2	95	65	2517	65	65	2517	2517	65	2517
S3	100	100	2406	105	100	2406	2406	100	2406
S4	84	59	2055	59	59	2055	2055	59	2055
S5	134	83	3593	83	83	3593	3593	83	3593
S6	111	47	5547	47	47	5547	5547	47	5547
S7	129	44	4612	44	44	4612	4612	44	4612
S8	123	67	3604	67	67	3604	3604	67	3604
S9	112	55	3733	55	55	3733	3733	55	3733
S10	106	56	3225	56	56	3225	3225	56	3225
Mean ± Std	104 ± 25	62 ± 18	3325 ± 1149	63 ± 18	62 ± 18	3325 ± 1149	3325 ± 1149	62 ± 18	3325 ± 1149

Similar to the approach taken in [5–7], we trained the classifier separately for each subject, and calculated the average of the AUCs and F1 scores for all subjects as the final result. For comparison purposes, the input data were identical for different classifiers. We focused on the performance of predicting subjects' braking intentions based on EEG at different moments prior to braking onset, for which we intercepted EEG segments on the test set through a sliding window of 1500 ms in length, and the endpoints of these intercepted segments varied in steps of 50 ms from −500 ms to 0 relative to the onset of braking. The intercepted data for testing used the same baseline correction method as the training data. Training and testing were performed on a computer with an Intel i7-7700 CPU, 32 GB of RAM, and NVIDIA 2080-Ti graphics card. The relevant settings of algorithms were as follows.

- For the CSP + LDA, we used the official library of MNE [33], while for the RMDM and TS + LR algorithms, we used the official library of pyRiemann [9]. No modifications were made to the three algorithms mentioned above, but the default parameter settings were used.
- For the xDAWN + LDA algorithm, we used the automatic shrinkage technique for the LDA classifier. Related studies have shown that the shrinkage LDA classifier outperforms the standard LDA classifier in EEG classification tasks [27, 34, 35]. The implementation of the xDAWN + LDA algorithm adopted the official library of pyRiemann.
- For the implementation of EEGNet and ShallowConvNet, we adopted the PyTorch framework and used the official library of braindecode [11]. The Adam optimizer was chosen and the weight-decay was set to 0.001, which could alleviate the overfitting problem by imposing a certain limit on the learning weights. The batch size was set to 32. The algorithm was run for 300 training iterations and the model with the lowest training loss was used as the test model.

### Evaluation metrics

Since the number of emergency braking epochs was much less than that of normal driving epochs, the classification accuracy could no longer be used to measure the performance of the algorithm. We used the area under the receiver operating characteristic curve (AUC) as a performance metric. The AUC takes values in the range [0, 1], with 0 representing complete incorrect classification, 1 representing completely correct classification, and 0.5 representing random classification [36]. The AUC gives an overall evaluation metric for binary classification problems with class imbalance, but it does not accurately reflect the recall and precision of the target class. In this experiment, the F1 score was also used as an evaluation metric because it took into account both precision and recall [37].

## Data Availability

The data and codes generated in this study can be obtained by emailing to the author (Xinbin Liang; lxb2203@163.com).
